# Credentialing of small‐field dosimetry for the lung SBRT in Japan Clinical Oncology Group clinical trial

**DOI:** 10.1002/acm2.70552

**Published:** 2026-04-15

**Authors:** Daisuke Kawahara, Shuichi Ozawa, Toshiyuki Minemura, Yu Kumazaki, Hideaki Hirashima, Satoshi Kito, Hiroyuki Okamoto, Mitsuhiro Nakamura, Teiji Nishio, Ikuno Nishibuchi, Yukinori Matsuo, Tomoki Kimura, Satoshi Ishikura, Naoto Shikama, Yasushi Nagata, Takashi Mizowaki

**Affiliations:** ^1^ Department of Radiation Oncology Graduate school of Biomedical and Health Sciences Hiroshima University Hiroshima Japan; ^2^ Hiroshima High‐Precision Radiotherapy Cancer Center Hiroshima Japan; ^3^ Section of Radiation Safety and Quality Assurance National Cancer Center Hospital Tokyo Japan; ^4^ Department of Radiation Oncology International Medical Center Saitama Medical University Saitama Japan; ^5^ Department of Radiation Oncology and Image‐Applied Therapy Kyoto University, Shogoin, Sakyo‐ku Kyoto Japan; ^6^ Department of Radiation Oncology, Department of Radiology Tokyo Metropolitan Cancer and Infectious Disease Center Komagome Hospital Tokyo Japan; ^7^ Radiation Safety and Quality Assurance Division National Cancer Center Hospital Tokyo Japan; ^8^ Department of Advanced Medical Physics, Graduate School of Medicine Kyoto University Kyoto Japan; ^9^ Medical Physics Laboratory, Division of Health Science, Graduate School of Medicine The University of Osaka Osaka Japan; ^10^ Department of Radiation Oncology Kindai University Faculty of Medicine, 377‐2, Ohno‐Higashi, Osaka‐Sayama Osaka Japan; ^11^ Department of Radiation Oncology Kochi Medical School Kochi University, Oko‐cho, Nangoku‐shi KohasuKochi Japan; ^12^ Department of Radiation Oncology St. Luke's International Hospital, St. Luke's International University, Chuo‐ku Tokyo Japan; ^13^ Department of Radiation Oncology Juntendo University Graduate School of Medicine Tokyo Japan; ^14^ Department of Radiation Oncology Chugoku Rosai Hospital, Emeritus Professor, Hiroshima University Kure Japan

**Keywords:** credentialing procedure for clinical trial, small field dosimetry, stereotactic body radiation therapy

## Abstract

**Background:**

Small‐field dosimetry plays a pivotal role in stereotactic body radiation therapy (SBRT), where high‐precision dose delivery is essential. In multi‐institutional clinical trials, quality assurance (QA) becomes increasingly critical due to the variability in linear accelerator (linac) output calibration and treatment planning system (RTPS) configurations. Conventional relative dose factor, which assess relative dose outputs, lack absolute dose information and thus cannot verify the consistency of linac calibration or monitor unit (MU)‐to‐dose conversion across institutions. These limitations may compromise treatment quality and undermine the integrity of clinical trials. Therefore, an enhanced QA method that incorporates absolute dosimetry is required.

**Purpose:**

This study introduces and implements a novel credentialing procedure for small‐field dosimetry in SBRT clinical trials using the absorbed dose per MU, expressed in cGy/MU. By integrating absolute dose information into the verification process, this method aims to evaluate both linac output calibration and small‐field modeling accuracy across institutions.

**Methods:**

A nationwide dosimetric survey was conducted involving 545 beams from 120 institutions participating in Japan Clinical Oncology Group (JCOG) trials. Treatment plans were created in each institution's RTPS by prescribing 10 Gy to the isocenter for square field sizes of 2 × 2 to 10 × 10 cm^2^. Doses were calculated at depths of 5 and 10 cm in a virtual water phantom under a fixed source‐to‐surface distance of 90 cm. The absorbed dose per MU values were calculated and compared against multicenter averages stratified by linac type and beam energy. A ±3% tolerance criterion was applied for credentialing. Institutions with deviations were contacted and corrective actions were taken as needed.

**Results:**

Of the 545 beams analyzed, 97.8% met the ±3% tolerance criteria. Discrepancies in the ten beams were attributed to small‐field beam modeling errors or incorrect RTPS MU‐to‐dose settings, which were subsequently corrected through feedback. Although variations were observed between the dose calculation algorithms and RTPSs, the differences were within 0.6%, 0.4%, and 1.6%, respectively. The credentialing process proved robust in standardizing dose calculations for clinical trial participation.

**Conclusion:**

The proposed absorbed dose per MU‐based method enables comprehensive small‐field dosimetry evaluation by incorporating absolute dose verification into the credentialing process. This approach effectively identifies calibration inconsistencies that are not detectable using traditional relative dose factor methods. Its application across a large cohort of institutions demonstrated high accuracy and inter‐institutional consistency, reinforcing its utility as a practical QA tool in SBRT clinical trials. By enhancing standardization in RTPS performance and linac output verification, this method strengthens the foundation of safe and reliable SBRT implementation in multi‐center clinical research.

## 1 INTRODUCTION

Stereotactic body radiation therapy (SBRT) is a cornerstone of modern radiotherapy for early‐stage non‐small cell lung cancer and has recently expanded into the treatment of oligometastatic disease. Its high precision relies on the integration of imaging, planning, and beam delivery techniques, underscoring the importance of QA procedures throughout the radiotherapy process.^1^ Since physics QA procedures are widely known to affect the clinical outcome of multi‐institutional clinical trials,^2^, ^3^, ^4^ they are essential for maintaining the quality of radiotherapy‐related clinical trials.

Globally, several multi‐institutional trials have also emphasized the role of dosimetry audits in ensuring the consistency of dose delivery. For example, the Radiation Therapy Oncology Group (RTOG) and its successor, NRG Oncology, and the TROG (Trans‐Tasman Radiation Oncology Group) in Australia and New Zealand have implemented stringent QA measures in their SBRT trials, including the use of anthropomorphic phantoms to verify the accuracy of dose delivery and small‐field dosimetry. These audits assess key parameters such as output factors (OF) and dose per MU calibration.^4^

In other multi‐institutional studies, the calculation accuracy of small fields, which is essential for SBRT, has been extensively investigated.^5^, ^6^, ^7^, ^8^ In conventional multi‐institutional dosimetric surveys and credentialing audits for SBRT, relative dose metrics such as output factors or relative dose factors are most commonly used to evaluate small‐field dosimetry. These relative evaluations are attractive because they are straightforward to implement and largely independent of absolute beam output calibration, allowing institutions with different calibration practices to be compared on a common relative basis. However, the exclusive reliance on relative metrics implicitly assumes that the absolute beam output calibration and the monitor‐unit–to–dose conversion in each institution's treatment planning system are correct. As a result, discrepancies in reference‐field output calibration or inconsistencies between linac calibration and radiotherapy treatment planning system (RTPS) modeling cannot be detected by relative‐only audits.

To confirm the consistency of the RTPS, including absolute dose information, it is necessary to check the relationship between linac output and OF. Although the use of postal dosimetry audits of high‐energy radiotherapy photon beams by a third party using thermoluminescent dosimeter, optical stimulated luminescence dosimeter, or glass dosimeter^9^, ^10^ has become widespread, the consistency of the linac output of the RTPS is not always confirmed therein. In other words, third‐party audit organizations sometimes focus on a particular procedure and omit confirming procedural consistency through the whole chain of radiotherapy process. To address this limitation, we propose the use of absorbed dose per MU as a credentialing metric. Unlike relative‐only evaluations, absorbed dose per MU simultaneously constrains the absolute beam output under reference conditions and the relative dose modeling for small‐fields. This dual constraint enables identification of calibration or modeling inconsistencies that would remain invisible in conventional relative‐dose‐based credentialing, representing a fundamental enhancement rather than an incremental modification of existing QA practices. For example, the mailing audit of linac output needs to check the constancy of dose per MU calibration of linac and RTPS.

The Japan Clinical Oncology Group–Radiation Therapy Study Group (JCOG‐RTSG) has conducted Phase I and Phase II trials on SBRT for non‐small cell lung cancer (JCOG0403 and JCOG0702),^11^, ^12^ while its Medical Physics Working Group (MPWG) reported a heterogeneity correction survey.^8^, ^9^ The JCOG1408 phase III clinical trial began,^13^, ^14^ and an image‐guided radiotherapy postal audit requiring all participating institutions and the on‐site or postal audit requiring the institutions that use IMRT have been implemented by the MPWG.^15^, ^16^, ^17^ Additionally, recent studies, such as JCOG2108 and JCOG2110, have extended SBRT applications to oligometastatic disease, further underscoring the importance of robust QA protocols in clinical trials.^18^, ^19^ In the JCOG1408, JCOG2108, and JCOG2110 clinical trials, institutions using IMRT verified the absolute dose consistency of their RTPS through dedicated IMRT audits.^16^, ^17^ In contrast, for institutions relying solely on three‐dimensional conformal radiotherapy (3D‐CRT), absolute dose consistency in the RTPS was not comprehensively evaluated, leaving a critical gap in QA. Because many institutions calibrate linac output to 1 cGy/MU under an SAD reference setup, absolute dose consistency can be assessed by directly comparing absorbed dose per MU against multicenter reference values. Therefore, we propose an innovative credentialing method that integrates absolute dosimetry with small‐field output verification using absorbed dose per MU, enabling a more comprehensive evaluation than relative dose factor alone.

This study presents a new approach to credentialing that addresses the shortcomings of current QA procedures by establishing a direct connection between absolute dose accuracy and small‐field OF evaluations. By implementing the proposed absorbed dose per MU method, we seek to enhance the precision and reliability of multi‐institutional SBRT clinical trials, addressing critical challenges in radiotherapy QA for clinical trials.

## 2 MATERIALS AND METHODS

### 2.1 Definition of the output factors

The OF generally used for small‐field dosimetry evaluation, relative dose factor, are defined as Equation (1), the ratio of the point dose at depth *d* for a field size (FS) normalized to the dose at the same depth for the reference field. The reference FS is usually 10 × 10 cm^2^. The point doses are calculated under same MU values for each FS on RTPS.

(1) $$g-OF\left(FS,\;d\right)=\frac{D\left(FS,d\right)}{D\left(FS_{ref},d\right)}\;$$

On the other hand, the absorbed dose per MU is defined as the point dose at depth *d* for the FS divided by the same MU delivered. The relationship between absorbed dose per MU and relative dose factor is expressed as following.

(2) $$\begin{array}{cc} a-OF\left(FS,\;d\right) & =\frac{D\left(FS,d\right)}{MU}=g-OF\left(FS,\;d\right)\\ & \;\times \frac{D\left(FS_{ref},d\right)}{MU} \end{array}$$

Absorbed dose per MU inherently includes the absolute dose calibration component, namely, the dose per MU under the 10 × 10 cm^2^ reference condition at the same depth d. In this study, the relative dose factor at each depth was normalized to unity by definition (g‐OF(10 × 10, 5 cm) = 1 and g‐OF(10 × 10, 10 cm) = 1). Consequently, dividing absorbed dose per MU by the relative dose factor yields the reference‐field dose per MU at depth d, as described in Equation (3).

(3) $$\frac{a-OF\left(FS,\;d\right)}{g-OF\left(FS,\;d\right)}=\frac{D\left(FS_{ref},d\right)}{MU}\;$$

### 2.2 Data collection

A survey of small‐field dosimetry was conducted by the Medical Physics Working Group (MPWG) within the Japan Clinical Oncology Group Radiation Therapy Study Group (JCOG‐RTSG), including charter participants of JCOG1408, JCOG2108, and JCOG2110, as well as other collaborating institutions. Table 1 summarizes the multi‐institutional average values of the calculated dose ratios for each photon beam category. Beam data were collected from a total of 545 photon beams across participating institutions.

**TABLE 1:** Summary of the absorbed dose per MU at a depth of 5 cm expressed as mean and standard deviation of the mean, grouped by treatment machine.

		Field size (cm)				
Linac model	Number of linacs	2 × 2	3 × 3	4 × 4	5 × 5	10 × 10
Varian_4 MV	28	0.817 ± 0.001	0.852 ± 0.001	0.880 ± 0.001	0.905 ± 0.000	0.987 ± 0.001
Varian_6 MV	127	0.846 ± 0.001	0.886 ± 0.001	0.915 ± 0.001	0.938 ± 0.001	1.010 ± 0.000
Varian_10 MV	89	0.866 ± 0.001	0.930 ± 0.001	0.965 ± 0.001	0.989 ± 0.000	1.060 ± 0.000
Varian_6 MV FFF	93	0.852 ± 0.001	0.888 ± 0.001	0.915 ± 0.001	0.936 ± 0.000	0.996 ± 0.000
Varian_10 MV FFF	78	0.907 ± 0.001	0.963 ± 0.001	0.991 ± 0.001	1.010 ± 0.001	1.050 ± 0.001
Elekta_4 MV	13	0.830 ± 0.002	0.868 ± 0.002	0.897 ± 0.002	0.919 ± 0.002	0.994 ± 0.001
Elekta_6 MV	46	0.865 ± 0.001	0.905 ± 0.001	0.932 ± 0.001	0.952 ± 0.001	1.020 ± 0.001
Elekta_10 MV	34	0.872 ± 0.002	0.934 ± 0.001	0.967 ± 0.001	0.989 ± 0.001	1.060 ± 0.001
Elekta_6 MV FFF	22	0.892 ± 0.002	0.932 ± 0.002	0.957 ± 0.001	0.973 ± 0.001	1.020 ± 0.002
Elekta_10 MV FFF	15	0.916 ± 0.002	0.976 ± 0.002	1.010 ± 0.002	1.020 ± 0.002	1.060 ± 0.002

The survey was based on dose calculations performed using institutional radiotherapy treatment planning systems (TPSs). The calculation conditions were standardized across institutions. A virtual water phantom (density 1.0 g/cm^3^) was generated within each TPS. The source‐to‐surface distance (SSD) was fixed at 90 cm, and this geometry was maintained consistently throughout the study. The linac isocenter (IC) was positioned at a depth of 10 cm. Point doses were calculated on the central beam axis at depths of 5 and 10 cm. Square field sizes of 2 × 2 cm^2^, 3 × 3 cm^2^, 4 × 4 cm^2^, 5 × 5 cm^2^, and 10 × 10 cm^2^ were evaluated. Field apertures were defined using both the jaws and the multileaf collimator (MLC) according to institutional beam modeling practice. For small field sizes, both jaws and MLC settings were adjusted to match the prescribed square dimensions. Absorbed dose per MU was then derived at both calculation depths. Consequently, each photon beam yielded ten absorbed dose per MU data points (five field sizes evaluated at two depths).

The calculation grid size was required to be ≤2.5 mm. For Monte Carlo–based calculations, a mean statistical uncertainty of ≤1% was required. All calculated beam data were subsequently stratified according to linac manufacturer (Varian or Elekta) and TPS model. The TPSs analyzed for Varian linacs included Eclipse, RayStation, Pinnacle, XiO, and Monaco, whereas those for Elekta linacs included Eclipse, RayStation, Pinnacle, and Monaco.

### 2.3 Credentialing

An absorbed dose per MU, expressed in units of cGy/MU, was calculated by each participating institution using its own RTPS and commissioned beam model. Multicenter averages were used as reference values for each beam category. Standard deviations were calculated to describe inter‐institutional variability but were not used to define credentialing tolerance levels. A fixed ±3% tolerance relative to the multicenter reference was applied as the pass/fail criterion. The percentage difference between the multicenter average and the institution's value as follows:

(4) $$\begin{array}{cc} & Percentage\;Difference\\ & \;=\frac{OF_{institution}-OF_{multicenter\;average}}{OF_{multicenter\;average}}\;\times 100 \end{array}$$

As shown in Table 2, a total of 545 beams were used to calculate the multicenter average for each energy. At least five beams for the FSs of 2 × 2 cm^2^, 3 × 3 cm^2^, 4 × 4 cm^2^, 5 × 5 cm^2^, and 10 × 10 cm^2^ were required to calculate the multicenter average, as described in Equation (4). Data sets with fewer than four beams were excluded from the study and were instead followed up with on‐site measurements. After determining the reference values and the multicenter average, the actual credentialing procedures for small‐field dosimetry were initiated for all photon beam categories listed in Table 1. To pass the credentialing procedure, the percentage difference for all 10 data points which were the beams for the FSs of 2 × 2 cm^2^, 3 × 3 cm^2^, 4 × 4 cm^2^, 5 × 5 cm^2^, and 10 × 10 cm^2^ at 5 and 10 cm depths had to remain within ±3% of the multicenter average, a tolerance level selected in accordance with prior multi‐institutional lung SBRT dosimetry audits in which absolute dose agreement within ±3% has been reported as a practical and clinically meaningful threshold for credentialing in multi‐center clinical trials.^4^, ^20^ Any absorbed dose per MU value exceeding the ±3% tolerance triggered a formal investigation. The underlying causes of the deviation were analyzed, including potential issues related to beam modeling parameters, output calibration settings, or MU‐to‐dose conversion within the RTPS. The findings were communicated to the respective institution, and corrective actions were recommended to ensure compliance with the credentialing criterion.

**TABLE 2:** Summary of the absorbed dose per MU at a depth of 10 cm expressed as mean and standard deviation of the mean, grouped by treatment machine.

		Field size (cm)				
Linac model	Number of linacs	2 × 2	3 × 3	4 MV4	5 × 5	10 × 10
Varian_4 MV	28	0.561 ± 0.001	0.590 ± 0.001	0.615 ± 0.001	0.639 ± 0.001	0.728 ± 0.001
Varian_6 MV	127	0.607 ± 0.001	0.640 ± 0.000	0.665 ± 0.001	0.688 ± 0.000	0.771 ± 0.000
Varian_10 MV	89	0.661 ± 0.001	0.712 ± 0.000	0.742 ± 0.000	0.765 ± 0.000	0.840 ± 0.000
Varian_6 MV FFF	93	0.592 ± 0.000	0.622 ± 0.000	0.645 ± 0.000	0.665 ± 0.000	0.736 ± 0.000
Varian_10 MV FFF	78	0.674 ± 0.001	0.719 ± 0.001	0.743 ± 0.001	0.761 ± 0.000	0.812 ± 0.000
Elekta_4 MV	13	0.580 ± 0.002	0.611 ± 0.002	0.637 ± 0.001	0.659 ± 0.001	0.741 ± 0.001
Elekta_6 MV	46	0.629 ± 0.001	0.663 ± 0.001	0.688 ± 0.001	0.708 ± 0.001	0.783 ± 0.000
Elekta_10 MV	34	0.664 ± 0.002	0.712 ± 0.001	0.743 ± 0.001	0.765 ± 0.001	0.837 ± 0.001
Elekta_6 MV FFF	22	0.645 ± 0.001	0.679 ± 0.001	0.704 ± 0.001	0.721 ± 0.001	0.780 ± 0.001
Elekta_10 MV FFF	15	0.690 ± 0.001	0.739 ± 0.002	0.766 ± 0.002	0.782 ± 0.002	0.826 ± 0.001

### 2.4 Data analysis

The collected absorbed dose per MUs were decomposed into relative dose factors and output information as shown in Equation (2) to analyze the OF management in absorbed dose per MUs. The means and standard deviations (SD) of all data points that passed the credentialing procedure were calculated to assess the variability of the dose calculation in the center of the irradiation field. The failed data points were removed from the mean and SD analysis; thus, the final mean and SD values only include the data within the tolerances.

For Varian's linac, which had the largest number of beams, a significance test was conducted to analyze the impact of various factors, including the dose calculation algorithm (Anisotropic Analytical Algorithm [AAA] vs. Acuros XB [AXB] vs. Other) and RTPS differences (Eclipse vs. RayStation vs. Pinnacle vs. XiO vs. Monaco). The significance test employed a two‐tailed equal variance *t*‐test, with a significance level set at a *P*‐value of less than 0.05 to determine whether significant differences existed. The conditions with insufficient beam numbers were excluded to maintain statistical reliability.

## 3 RESULTS

**FIGURE 1:**
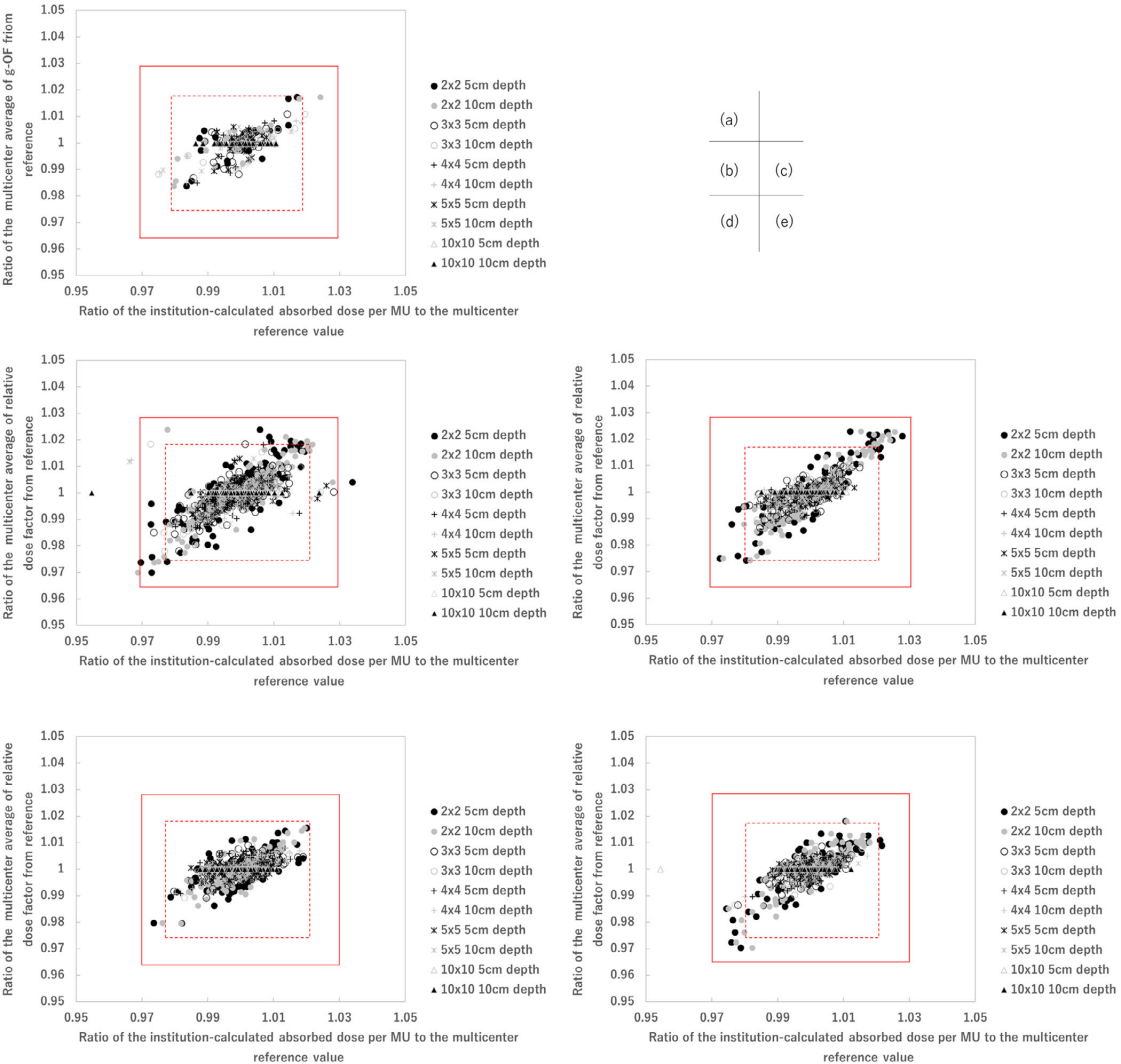
The absorbed dose per MU versus relative dose factor space plot of Varian 4 MV (a), 6 MV (b), 10 MV (c), 6 MV FFF (d), and 10 MV FFF (e) (415 beams).

Tables 1, 2, 3 summarize the absorbed dose per MUs at a depth of 5 and 10 cm and relative dose factors expressed as mean and standard deviation, grouped by treatment machine and FS. These tables provide the baseline reference values for the analysis. Based on these baseline values, a tolerance threshold of ±3% was used as the formal criterion for credentialing. Additionally, a ±2% range was analyzed for reference purposes to illustrate stricter variability. Of the 545 beams analyzed, 97.8% met the ±3% tolerance criteria. It should be noted that the ±2% range analyzed in this study is not the tolerance threshold but is used as a reference to illustrate stricter variability and provide additional insight into calculation consistency. Figure 1 presents the ratio of the multicenter average calculated and reference absorbed dose per MU at a depth of 5 and 10 cm and relative dose factor plot for Varian 4 MV (a), 6 MV (b), 10 MV (c), 6 MV FFF (d), and 10 MV FFF (e) (415 beams). A total of 12 beams were outside the 3% tolerance in Table 4. Among these, six beams were associated with Varian linacs. It was also observed that for the smallest FS of 2 × 2 cm^2^, data exhibited a larger overall spread across all energy levels. Many of these points were outside the dashed line region, representing the 2% range, indicating greater variability for this FS compared to larger fields. The variability observed for 6 MV FFF and 10 MV FFF beams was smaller compared to FF beams. The data points for FFF beams were more tightly clustered within or near the ±2% range, indicating higher inter‐institutional consistency of absorbed dose per MU values compared to FF beams. The analysis of absorbed dose per MUs and relative dose factors across various FSs, energy levels, and configurations revealed distinct trends in variability, as summarized in Tables 5, 6, 7. As shown in Table 5, the analysis of absorbed dose per MUs at 5 cm depth revealed that the percentage of data points exceeding the 2% and 3% thresholds was highest for the smallest FS (2 × 2 cm^2^) across all energy levels. For example, 7.1% of data for 4 MV exceeded the 2% threshold, with 3.6% exceeding 3%. Similarly, for 10 MV, 15.4% of data points exceeded 2%, although only 0.8% exceeded 3%. The FFF beams, such as 6 MV FFF and 10 MV FFF, exhibited lower variability, with no data points exceeding the 3% threshold at any FS. As shown in Table 6, the variability was the same trend at 10 cm depth. The percentage of data points exceeding 2% was highest for 4 MV. On the other hand, the percentage of data points exceeding 3% was within 3.6% for all energies. Table 7 shows the percentage of data points exceeding the 2% and 3% tolerance thresholds for the relative dose factor. It showed lower overall variability compared to absorbed dose per MUs. Across all energy levels and configurations, the relative dose factor data showed a maximum of 9.8% exceeding the 2% threshold (10 MV, 2 × 2 cm^2^), and 0.8% exceeding the 3% threshold (6 MV).

**TABLE 3:** Summary of the relative dose factor expressed as mean and standard deviation of the mean, grouped by treatment machine.

		Field size (cm)			
Linac model	Number of linacs	2 × 2	3 × 3	4 × 4	5 × 5
Varian_4 MV	28	0.771 ± 0.001	0.810 ± 0.001	0.845 ± 0.001	0.878 ± 0.000
Varian_6 MV	127	0.787 ± 0.001	0.830 ± 0.000	0.863 ± 0.000	0.892 ± 0.000
Varian_10 MV	89	0.786 ± 0.001	0.848 ± 0.000	0.883 ± 0.000	0.910 ± 0.000
Varian_6 MV FFF	93	0.805 ± 0.000	0.845 ± 0.000	0.877 ± 0.000	0.904 ± 0.000
Varian_10 MV FFF	78	0.831 ± 0.001	0.886 ± 0.000	0.915 ± 0.000	0.937 ± 0.000
Elekta_4 MV	13	0.782 ± 0.002	0.824 ± 0.001	0.859 ± 0.001	0.889 ± 0.001
Elekta_6 MV	46	0.802 ± 0.001	0.846 ± 0.001	0.878 ± 0.000	0.904 ± 0.000
Elekta_10 MV	34	0.793 ± 0.002	0.851 ± 0.001	0.887 ± 0.001	0.914 ± 0.001
Elekta_6 MV FFF	22	0.827 ± 0.001	0.871 ± 0.001	0.903 ± 0.001	0.924 ± 0.000
Elekta_10 MV FFF	15	0.836 ± 0.001	0.894 ± 0.001	0.926 ± 0.001	0.946 ± 0.001

**TABLE 4:** Summary of beams exceeding the 3% tolerance threshold, including the corresponding linacs, specific configurations, and corrective actions provided to address identified discrepancies.

Machine	Energy	Number of errors	Corrective actions
Clinac 21EX	6	1	Comparison of the measurement and calculation
Clinac iX	6	1	Re‐commissioning
TrueBeam	6	1	Comparison of the measurement and calculation
TrueBeam	6	3	Comparison of the measurement and calculation
Synergy	10	2	Comparison of the measurement and calculation
Agilitygy	10	2	Comparison of the measurement and calculation

**TABLE 5:** Percentage of data points exceeding the 2% and 3% tolerance thresholds for the a‐OFs at a depth of 5 cm across different field sizes, energy levels, and treatment machine configurations.

	>2%					>3%				
	Field size (cm)					Field size (cm)				
	2 × 2	3 × 3	4 MV4	5 × 5	10 × 10	2 × 2	3 × 3	4 MV4	5 × 5	10 × 10
4 MV	7.1%	7.1%	0.0%	0.0%	0.0%	3.6%	0.0%	0.0%	0.0%	0.0%
6 MV	8.1%	4.0%	1.2%	1.2%	0.6%	1.2%	0.0%	0.0%	0.0%	0.0%
10 MV	15.4%	0.0%	0.0%	0.0%	0.0%	0.8%	0.0%	0.0%	0.0%	0.0%
6 MV FFF	3.3%	1.1%	1.1%	0.0%	0.0%	0.0%	0.0%	0.0%	0.0%	0.0%
10 MV FFF	10.4%	1.5%	0.0%	0.0%	0.0%	0.0%	0.0%	0.0%	0.0%	0.0%

**TABLE 6:** Percentage of data points exceeding the 2% and 3% tolerance thresholds for the a‐OFs at a depth of 10 cm across different field sizes, energy levels, and treatment machine configurations.

	> 2%					> 3%				
	Field size (cm)					Field size (cm)				
	2 × 2	3 × 3	4 × 4	5 × 5	10 × 10	2 × 2	3 × 3	4 × 4	5 × 5	10 × 10
4 MV	17.9%	10.7%	7.1%	3.6%	0.0%	3.6%	0.0%	0.0%	0.0%	0.0%
6 MV	8.7%	3.5%	1.7%	1.2%	1.2%	0.6%	0.0%	0.6%	0.6%	0.6%
10 MV	10.6%	0.0%	0.0%	0.0%	0.0%	1.6%	0.0%	0.0%	0.8%	0.0%
6 MV FFF	2.2%	0.0%	2.2%	0.0%	0.0%	0.0%	0.0%	0.0%	0.0%	0.0%
10 MV FFF	6.0%	1.5%	1.5%	0.0%	0.0%	0.0%	0.0%	0.0%	0.0%	0.0%

**TABLE 7:** Percentage of data points exceeding the 2% and 3% tolerance thresholds for the g‐OF across different field sizes, energy levels, and treatment machine configurations.

	> 2%				> 3%			
	Field size (cm)				Field size (cm)			
	2 × 2	3 × 3	4 × 4	5 × 5	2 × 2	3 × 3	4 × 4	5 × 5
4 MV	3.6%	0.0%	0.0%	0.0%	0.0%	0.0%	0.0%	0.0%
6 MV	5.2%	0.6%	0.0%	0.0%	0.8%	0.8%	0.8%	0.8%
10 MV	9.8%	0.0%	0.0%	0.0%	0.0%	0.0%	0.0%	0.0%
6 MV FFF	2.2%	0.0%	0.0%	0.0%	0.0%	0.0%	0.0%	0.0%
10 MV FFF	3.7%	0.0%	0.0%	0.0%	0.0%	0.0%	0.0%	0.0%

**FIGURE 2:**
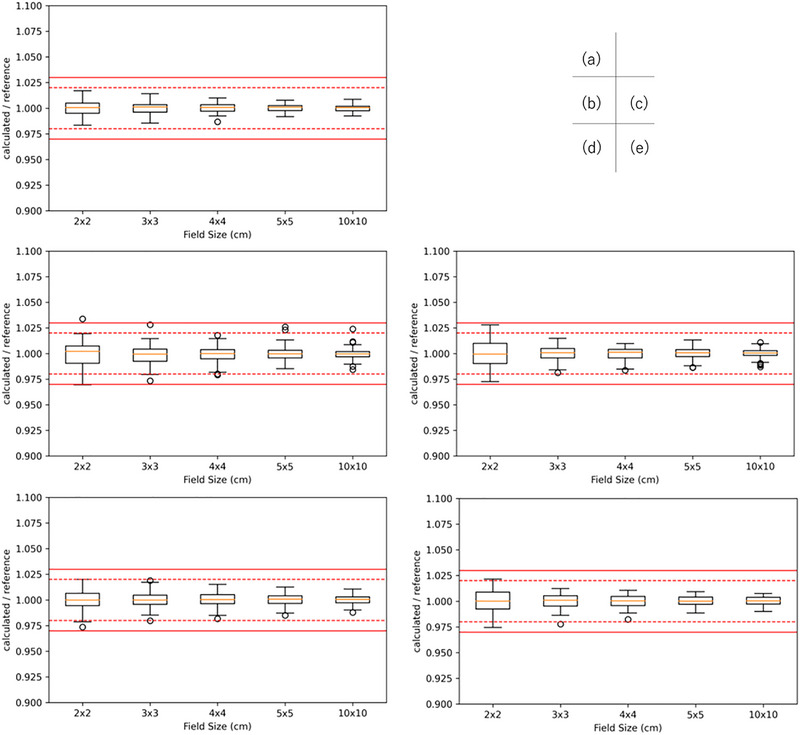
Box‐and‐whisker plots of absorbed dose per MU for Varian linacs at a calculation depth of 5 cm in water. Panels (a)–(e) correspond to 4 MV, 6 MV, 10 MV, 6 MV FFF, and 10 MV FFF, respectively. For each field size (2 × 2, 3 × 3, 4 × 4, 5 × 5, and 10 × 10 cm^2^), the box represents the interquartile range (IQR) with the median indicated by the horizontal line; whiskers extend to 1.5 × IQR, and points denote outliers. Red solid and dashed horizontal lines indicate the ±3% and ±2% tolerance bands, respectively.

**FIGURE 3:**
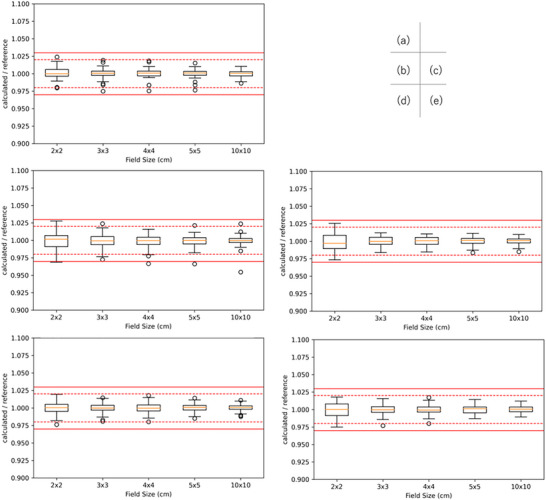
Box‐and‐whisker plots of absorbed dose per MU for Varian linacs at a calculation depth of 10 cm in water. Panels (a)–(e) correspond to 4 MV, 6 MV, 10 MV, 6 MV FFF, and 10 MV FFF, respectively. The box indicates the IQR with the median shown as a horizontal line; whiskers extend to 1.5×IQR, and points denote outliers. Red solid and dashed horizontal lines indicate the ±3% and ±2% tolerance bands, respectively, for visual assessment of the credentialing criteria.

**FIGURE 4:**
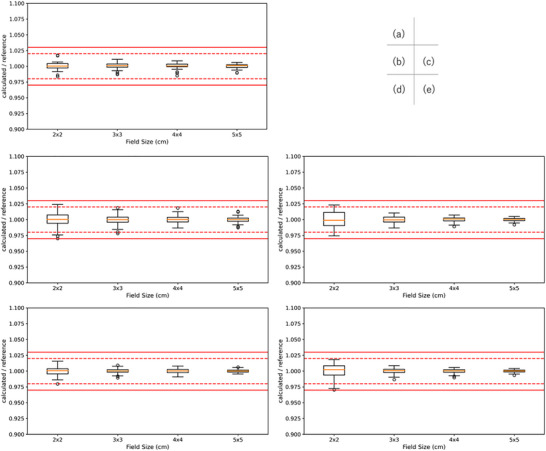
Box‐and‐whisker plots of relative dose factor, expressed as the ratio of TPS‐calculated values to the corresponding reference values, for square field sizes ranging from 2 × 2 to 5 × 5 cm^2^ for Varian linacs. The central line of each box indicates the median, the box represents the interquartile range, and the whiskers denote 1.5 times the interquartile range; open circles indicate outliers. The solid red lines indicate the ±3% credentialing tolerance, while the dashed red lines indicate the ±2% reference range. All observed distributions remained within the ±3% tolerance, demonstrating acceptable consistency of output factor modeling across institutions for small‐field SBRT applications.

Figures 2, 3, 4 show box‐and‐whisker plots of the ratio of institution‐calculated absorbed dose per MU to the corresponding multicenter reference value at a depth of 5 and 10 cm and relative dose factor for Varian linacs. For 6 MV, 6 data points exceeded the 3% tolerance for absorbed dose per MU, corresponding to FSs of 1 point for 2 × 2 cm^2^, at a depth of 5 cm, 2 points for 2 × 2 cm^2^, 1 point for 4 × 4 cm^2^, 1 point for 5 × 5 cm^2^ and 1 point for 10 × 10 cm^2^ at a depth of 10 cm. On the other hand, for 10 MV, 6 MV FFF, and 10 MV FFF, all data points for both absorbed dose per MU and relative dose factor were within the 3% tolerance range, demonstrating consistent agreement across these energy levels.

**FIGURE 5:**
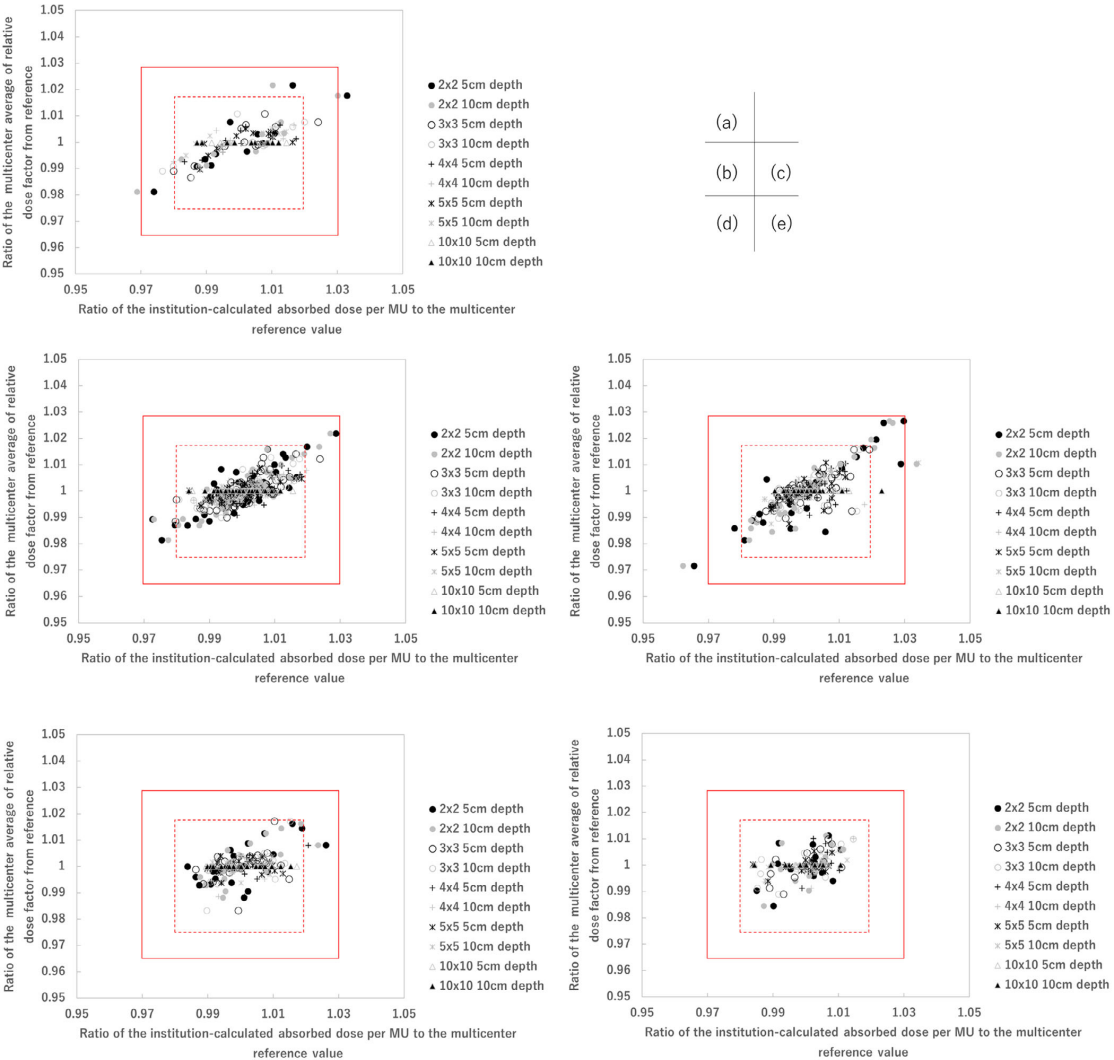
The absorbed dose per MU versus relative dose factor space plot of Elekta 4 MV (a), 6 MV (b), 10 MV (c), 6 MV FFF (d), and 10 MV FFF (e) (130 beams).

Figure 5 presents the absorbed dose per MU versus relative dose factor space plot of Elekta 4 MV (a), 6 MV (b), 10 MV (c), 6 MV FFF (d), and 10 MV FFF (e) (130 beams). A total of 12 beams for the outside, the 3% tolerance, 6 beams were Elekta linacs. It was also observed that for the smallest FS of 2 × 2 cm^2^, data exhibited a larger overall spread across all energy levels (Figure 5). Many of these points were outside the dashed line region, representing the 2% range, indicating greater variability for this FS compared to larger fields. The variability observed for 6 MV FFF and 10 MV FFF beams was smaller compared to FF beams. The data points for FFF beams were more tightly clustered within or near the 2% tolerance range, demonstrating higher consistency in calculation compared to FF beams.

**FIGURE 6:**
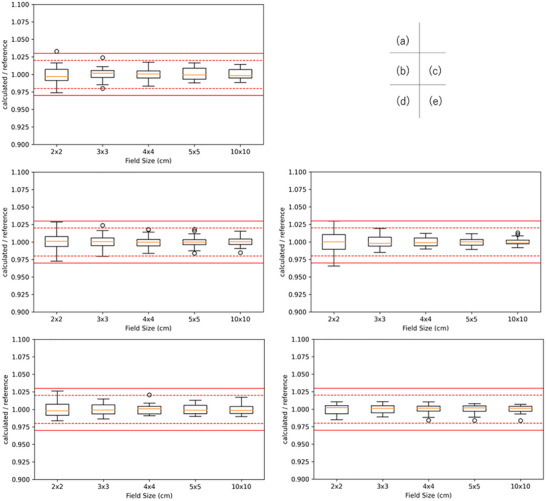
Box‐and‐whisker plots of absorbed dose per MU for Elekta linacs at a calculation depth of 5 cm in water. Panels (a)–(e) correspond to 4 MV, 6 MV, 10 MV, 6 MV FFF, and 10 MV FFF, respectively. For each field size (2 × 2, 3 × 3, 4 × 4, 5 × 5, and 10 × 10 cm^2^), the box represents the IQR with the median indicated by the horizontal line; whiskers extend to 1.5 × IQR, and points denote outliers. Red solid and dashed horizontal lines indicate the ±3% and ±2% tolerance bands, respectively.

**FIGURE 7:**
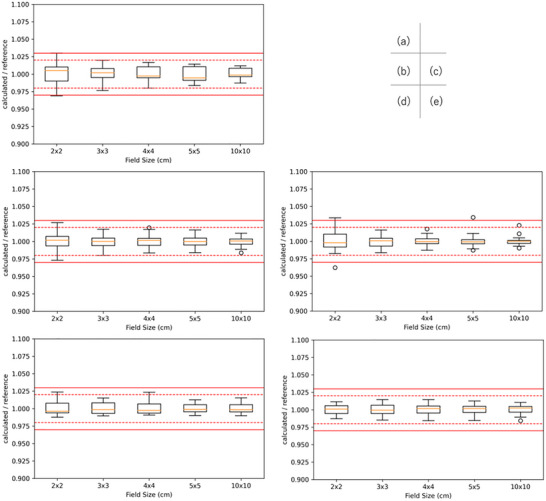
Box‐and‐whisker plots of absorbed dose per MU for Elekta linacs at a calculation depth of 10 cm in water. Panels (a)–(e) correspond to 4 MV, 6 MV, 10 MV, 6 MV FFF, and 10 MV FFF, respectively. The box indicates the IQR with the median shown as a horizontal line; whiskers extend to 1.5 × IQR, and points denote outliers. Red solid and dashed horizontal lines indicate the ±3% and ±2% tolerance bands, respectively, for visual assessment of the credentialing criteria.

**FIGURE 8:**
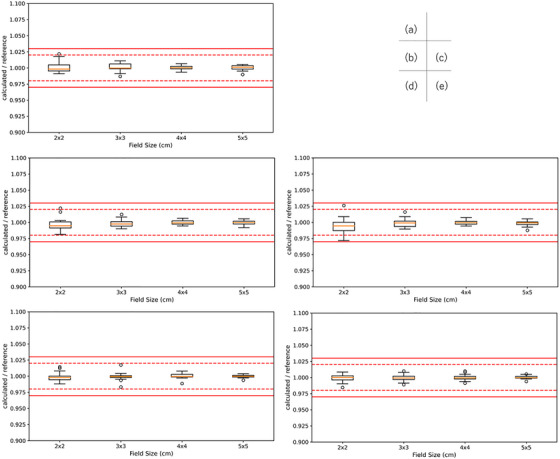
Box‐and‐whisker plots of relative dose factor, expressed as the ratio of TPS‐calculated values to the corresponding reference values, for square field sizes ranging from 2 × 2 to 5 × 5 cm^2^ for Elekta linacs. The central line of each box indicates the median, the box represents the interquartile range, and the whiskers denote 1.5 times the interquartile range; open circles indicate outliers. The solid red lines indicate the ±3% credentialing tolerance, while the dashed red lines indicate the ±2% reference range. All observed distributions remained within the ±3% tolerance, demonstrating acceptable consistency of output factor modeling across institutions for small‐field SBRT applications.

Figures 6, 7, 8 show box‐and‐whisker plots of the ratio of institution‐calculated absorbed dose per MU to the corresponding multicenter reference value at a depth of 5 and 10 cm and relative dose factor for Elekta linacs. For 4 MV, 2 data points exceeded the 3% tolerance for absorbed dose per MU, corresponding to FSs of 2 points for 2 × 2 cm^2^ at a depth of 5 and 10 cm. For 10 MV, 4 data points exceeded the 3% tolerance for absorbed dose per MU, corresponding to FSs of 1 point for 2 × 2 cm^2^ at a depth of 5 cm and 2 points at a depth of 10 cm. On the other hand, for 6 MV, 6 MV FFF, and 10 MV FFF, all data points for both absorbed dose per MU and relative dose factor were within the 3% tolerance range, demonstrating consistent agreement across these energy levels.

Additionally, the beams that exceeded the 3% tolerance threshold were investigated, and the corresponding linacs and their specific configurations are summarized in Table 4. This table also outlines the corrective actions provided to the respective institutions to address the identified discrepancies. Among the cases, only one beam underwent re‐commissioning to resolve the issue. For the remaining facilities, deviations were evaluated through review of RTPS configuration and beam modeling parameters. Agreement within the ±3% tolerance was confirmed by recalculation under the predefined virtual phantom conditions.

**FIGURE 9:**
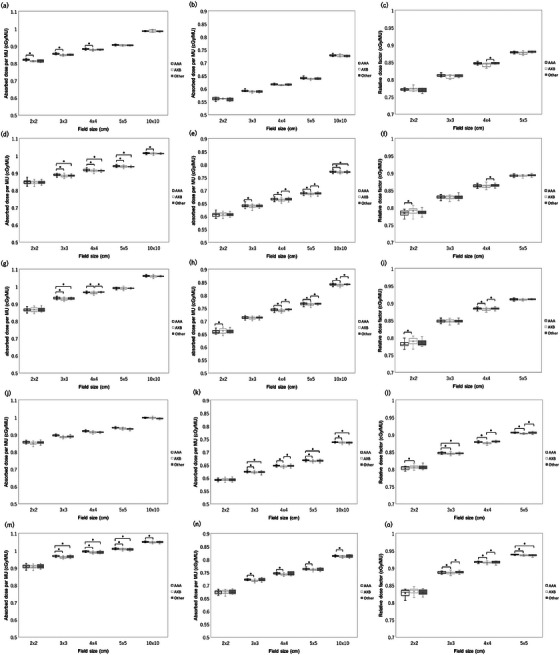
The comparison of RTPS (Eclipse vs. RayStation vs. Pinnacle vs. XiO vs. Monaco) for Varian linacs. The box plot shows the ratio of multicenter average calculated and reference absorbed dose per MU at a depth of 5 cm (a), 10 cm (b), and relative dose factor (c) for Varian 4 MV, at a depth of 5 cm (d), 10 cm (e), and relative dose factor (f) for Varian 6 MV, at a depth of 5 cm (g), 10 cm (h), and relative dose factor (i) for Varian 10 MV, at a depth of 5 cm (j), 10 cm (k), and relative dose factor (l) for Varian 6 MV FFF, and at a depth of 5 cm (m), 10 cm (n), and relative dose factor (o) for Varian 10 MV FFF for each RTPS.

**FIGURE 10:**
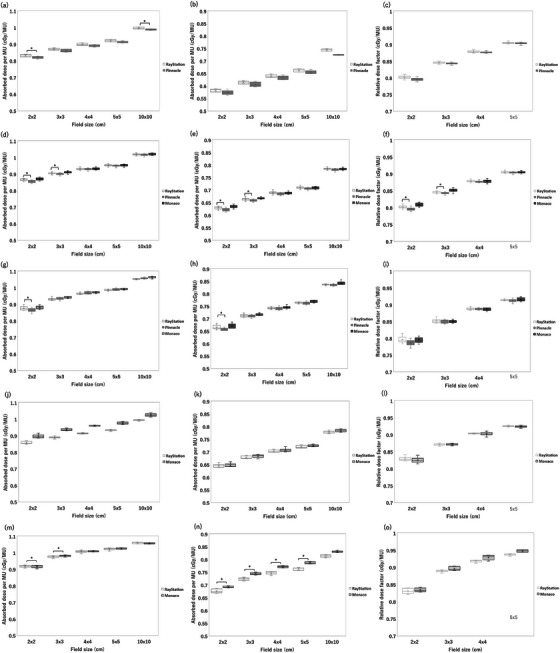
The comparison of RTPS (RayStation vs. Pinnacle vs. Monaco) for Elekta linacs. The box plot shows the ratio of multicenter average calculated and reference absorbed dose per MU at a depth of 5 cm (a), 10 cm (b), and relative dose factor (c) for Elekta 4 MV, at a depth of 5 cm (d), 10 cm (e), and relative dose factor (f) for Elekta 6 MV, at a depth of 5 cm (g), 10 cm (h), and relative dose factor (i) for Elekta 10 MV, at a depth of 5 cm (j), 10 cm (k), and relative dose factor (l) for Elekta 6 MV FFF, and at a depth of 5 cm (m), 10 cm (n), and relative dose factor (o) for Elekta 10 MV FFF for each RTPS.

Figure 9 shows the comparison of RTPS (Eclipse vs. RayStation vs. Pinnacle vs. XiO vs. Monaco) for Varian linacs. There was no institution that used XiO for 4X and 6 MV FFF. Significant differences were observed in a total of 45 cases. Specifically, significant differences were found in six cases of absorbed dose per MU at a depth of 5 cm, seven cases of absorbed dose per MU at a depth of 10 cm, and five cases in relative dose factor for the Eclipse‐RayStation comparison, four cases of absorbed dose per MU at a depth of 5 cm and one case of relative dose factor for the Eclipse‐Pinnacle comparison, four cases of absorbed dose per MU at a depth of 5 cm, and three cases of absorbed dose per MU at a depth of 10 cm for the Eclipse‐XiO comparison, five cases of absorbed dose per MU at a depth of 5 cm, four cases of absorbed dose per MU at a depth of 10 cm, and one cases in relative dose factor for the RayStation‐Pinnacle comparison, three cases of absorbed dose per MU at a depth of 5 cm and two cases of absorbed dose per MU at a depth of 10 cm for the Pinnacle‐XiO comparison. Among these, the Eclipse‐RayStation comparison exhibited the highest number of significant differences. The maximum dose difference was 0.9% for 4 MV with a 2 × 2 cm^2^ FS of absorbed dose per MU at a depth of 5 cm, 1.3% for 6 MV with a 2 × 2 cm^2^ FS of absorbed dose per MU at a depth of 5 cm, 1.0% for 10 MV with a 2 × 2 cm^2^ FS of absorbed dose per MU at a depth of 5 cm, 1.3% for 6 MVFFF with a 2 × 2 cm^2^ FS of absorbed dose per MU at a depth of 5 cm, and 1.6% for 10 MV FFF with a 2 × 2 cm^2^ FS of absorbed dose per MU at a depth of 5 cm. Figure 10 shows the comparison of RTPS models (RayStation vs. Pinnacle vs. Monaco) for Elekta linacs. No Elekta datasets using XiO were available for this analysis, and the number of Pinnacle datasets was insufficient for 6 MV FFF and 10 MV FFF; therefore, those FFF comparisons were limited to RayStation versus Monaco. Significant differences were observed in a total of 18 cases. Specifically, for the RayStation–Pinnacle comparison, significant differences were found in five cases for absorbed dose per MU at a depth of 5 cm, three cases for absorbed dose per MU at a depth of 10 cm, and two cases for the relative dose factor. For the RayStation–Monaco comparison, significant differences were identified in four cases for absorbed dose per MU at a depth of 5 cm and four cases for absorbed dose per MU at a depth of 10 cm. Among these, the RayStation–Pinnacle comparison exhibited the highest number of significant differences. The maximum dose difference was 1.4% for 4 MV with a 2 × 2 cm^2^ field size (absorbed dose per MU at 5 cm depth), 1.4% for 6 MV with a 2 × 2 cm^2^ field size (absorbed dose per MU at 5 cm depth), 1.8% for 10 MV with a 2 × 2 cm^2^ field size (absorbed dose per MU at 5 cm depth), 0.9% for 6 MV FFF with a 3 × 3 cm^2^ field size (absorbed dose per MU at 5 cm depth), and 0.8% for 10 MV FFF with a 3 × 3 cm^2^ field size.

## 4 DISCUSSION

This study presented a novel credentialing framework utilizing absorbed dose per MU, expressed in cGy/MU, to address limitations inherent in traditionally relative dose factor evaluations. The key innovation of this approach lies in its integration of absolute dose information into small‐field dosimetry, enabling the detection of deviations that would otherwise remain unnoticed in relative dose factor‐based evaluations. Although conventional relative dose factor evaluations did not indicate any deviations, the absorbed dose per MU approach revealed discrepancies that would otherwise have remained undetected, highlighting its added value for quality assurance.

By incorporating absolute dose consistency directly into the analysis, absorbed dose per MU enables a more comprehensive evaluation of linac output settings and RTPS beam modeling than relative dose factor alone. While relative dose factors effectively normalize relative dose variations across field sizes, they do not capture inconsistencies related to absolute dose calibration. In the present study, deviations identified using absorbed dose per MU were subsequently investigated. The underlying causes were attributed to small‐field output modeling and commissioning‐related factors, including differences in detector selection and correction, beam model parameterization, and jaw‐ or MLC‐defined field aperture modeling within the TPS. After targeted on‐site investigations and beam re‐modeling, recalculated dose values fell within ±2% of the multicenter average, confirming resolution of the discrepancies. These observations indicate that the detected deviations were associated with modeling and calibration processes rather than intrinsic linac performance. Overall, the results demonstrate that absorbed dose per MU can serve as a useful indicator of potential inconsistencies in absolute dose calibration during commissioning, thereby providing an additional layer of QA oversight that complements conventional relative dose–based credentialing approaches.

This reflects the fact that linac output adjustment is not always performed with explicit consideration of consistency with the treatment planning system, and in some facilities, it may be adjusted solely to achieve 1 cGy/MU at the depth of maximum dose on the linac, independent of the treatment plan configuration. Under AAPM TG‐51, beam quality (PDD10x) is defined using a 10 × 10 cm^2^ field at SSD = 100 cm in water. However, some institutions perform absolute dose calibration under alternative geometries, such as SSD = 90 cm or SAD‐based setups, depending on local policy. In the United States, the choice between SSD and SAD reference geometry for beam output calibration is determined at the institutional level. Differences in reference geometry can introduce predictable scaling effects due to inverse‐square relationships and depth‐dependent factors. Therefore, when institutions employ different calibration geometries, appropriate harmonization or geometry‐specific conversion coefficients are required to enable unbiased comparison under a unified reference condition. Because RTPS commissioning is based on measured beam data acquired under the institution's chosen calibration geometry, absorbed dose per MU calculated within the RTPS inherently reflects those calibration practices, even though the present credentialing framework is based on standardized calculation conditions.

The utility of absorbed dose per MU was evaluated against findings from previous international audits, particularly those focusing on small‐field dosimetry. Lechner et al. conducted a multinational audit of small‐field OPF, reporting significant variability among institutions and RTPS. By contrast, the present study demonstrated substantially improved inter‐institutional consistency when absorbed dose per MU was used as the credentialing metric, even for the smallest FSs. This dramatic reduction highlights the improved consistency achieved through the integration of absolute dose calibration. In recent years, the commissioning of the RTPS using standardized beam data, such as Varian's representative beam data,^10^ has become increasingly common. This practice has contributed to the unification of beam models, even across general‐purpose linear accelerators, leading to improved consistency in treatment planning system performance.

Differences among treatment planning systems were observed in small‐field dose calculations, particularly for the smallest FSs.^21^ Such RTPS‐dependent variability in small‐field dosimetry has been consistently reported in previous studies, reflecting the inherent challenges in accurately modeling dose distributions under small‐field conditions. For example, Kawahara et al. reported substantial algorithm‐dependent differences in output factors for very small‐fields using flattening filter‐free beams,^22^ while Bosse et al. demonstrated notable discrepancies among Monaco, Pinnacle, and Eclipse treatment planning systems, especially as FS decreased and in the presence of tissue inhomogeneities.^23^ In the present study, although statistically significant differences among RTPSs were identified, all observed discrepancies remained within the ±3% credentialing tolerance. These results indicate that, under the homogeneous water phantom conditions used in this study, the observed variations in TPS beam model implementation are unlikely to affect SBRT trial credentialing outcomes, as all differences were within the predefined tolerance band. Nevertheless, the observed variability underscores the importance of comprehensive credentialing to ensure consistent dose modeling across institutions, particularly for small‐field SBRT applications.

The observed discrepancies in dose calculations for small FSs, particularly for the smallest fields and shallower depths, can be attributed to commissioning practices and beam modeling methodologies rather than intrinsic limitations of the linac. Small‐field dosimetry is well known to be sensitive to detector limitations, partial volume effects, and algorithm‐dependent modeling assumptions, all of which can lead to increased uncertainty under these conditions. In clinical commissioning, measurements are often prioritized at standard FSs and depths, typically around a 10 × 10 cm^2^ field at a reference depth, to establish baseline beam data.^24^ As a result, dose accuracy for very small‐fields or at shallower depths may receive less rigorous validation during commissioning. For example, vendor recommendations often discourage the use of measured data from extremely small‐fields because of detector‐related uncertainties, which may contribute to the discrepancies observed in this study. International guidance has emphasized the importance of comprehensive commissioning across a range of FSs and depths to ensure accurate dose modeling in treatment planning systems.^25^ Taken together, these considerations suggest that the discrepancies identified in small‐field dose calculations reflect known challenges in commissioning and beam modeling, and further support the value of credentialing approaches that can sensitively detect such effects in a multi‐institutional setting. Building on these findings, this study introduced the evaluation of absorbed dose per MU as a credentialing tool for assessing inter‐institutional RTPS dose calculations in preparation for multicenter SBRT clinical trials, such as JCOG1408, JCOG2108, and JCOG2110. By implementing absorbed dose per MU assessments, discrepancies in absolute dose calibration across participating institutions were identified and addressed, enabling more consistent and accurate dose modeling prior to trial initiation. Beam output calibration geometry represents an important consideration in interpreting absorbed dose per MU comparisons. In principle, systematic differences may arise between SAD‐based and SSD‐based reference calibration due to inverse‐square effects under a fixed reference geometry. Such differences could introduce predictable scaling factors when absorbed dose per MU values are compared without accounting for calibration geometry. In the present dataset, however, the majority of participating institutions calibrated their linac output under SAD‐based reference conditions consistent with national practice. Therefore, large systematic offsets attributable to calibration geometry were not observed across the cohort. The cluster identified by the reviewer likely reflects institution‐specific calibration or beam modeling characteristics rather than a mixed‐geometry effect across the entire dataset. We acknowledge that strict inter‐institutional standardization of reference calibration geometry would further strengthen the robustness of this credentialing framework. For future implementations involving heterogeneous calibration geometries, application of geometry‐specific correction factors or explicit harmonization of reference conditions would be advisable to ensure unbiased comparison.

The present framework is not intended to replace independent third‐party phantom audits. Rather, it functions as a complementary credentialing tool that evaluates internal consistency between beam output calibration and RTPS modeling under standardized calculation conditions. External audits remain essential for end‐to‐end verification of dose delivery, whereas absorbed dose per MU specifically targets systematic discrepancies in small‐field beam modeling and MU‐to‐dose consistency. Although the present study implemented this approach as a credentialing procedure for clinical trial participation, the methodology is not inherently limited to one‐time evaluation. The absorbed dose per MU framework may also be applied longitudinally, for example following beam model updates, TPS software upgrades, or output recalibration, to ensure continued consistency over time.

## 5 CONCLUSION

This study introduced a novel credentialing framework utilizing absorbed dose per MU expressed in cGy/MU to address limitations in traditional small‐field dosimetry evaluations. By incorporating absolute dose information directly into OF measurements, the absorbed dose per MU approach enables a more comprehensive evaluation of linac output settings and radiotherapy treatment planning system (RTPS) performance. This framework effectively identified discrepancies in dose calibration and small‐field dosimetry that would otherwise remain undetected using conventional relative dose factor. The results demonstrated that the proposed method provides a reliable and robust quality assurance (QA) approach, achieving consistent agreement within a 3% tolerance relative to multicenter averages. By linking absolute dose consistency with small‐field OF evaluations, the proposed method enhances the precision and reliability of stereotactic body radiation therapy (SBRT) across multiple institutions. This approach ensures more accurate and standardized dose delivery, thereby improving the overall quality of radiotherapy treatments and contributing to better clinical outcomes in multi‐institutional trials. Future implementation of this framework could further refine QA protocols, supporting advancements in radiotherapy dosimetry and treatment planning system validation.

## AUTHOR CONTRIBUTIONS

Daisuke Kawahara and Shuichi Ozawa conceived and designed the study. Daisuke Kawahara, Shuichi Ozawa, Toshiyuki Minemura, Yu Kumazaki, Hideaki Hirashima, Satoshi Kito, and Hiroyuki Okamoto contributed to data acquisition and quality assurance. Y.Mu., Ikuno Nishibuchi, Yukinori Matsuo, Tomoki Kimura, Satoshi Ishikura, Naoto Shikama, Yasushi Nagata, and Takashi Mizowaki contributed to clinical implementation and data interpretation. Mitsuhiro Nakamura and T.N. provided overall supervision of the clinical trial regarding medical physics aspects. Y.Mu., Ikuno Nishibuchi, Yukinori Matsuo, Tomoki Kimura, Satoshi Ishikura, Naoto Shikama, Yasushi Nagata, and Takashi Mizowaki provided overall supervision of the clinical trial regarding clinical aspects. Daisuke Kawahara and Shuichi Ozawa drafted the manuscript. All authors critically reviewed the manuscript, contributed to the interpretation of the results, and approved the final version.

## CONFLICT OF INTEREST STATEMENT

The authors declare no conflicts of interest.
